# A Single Crystal Hybrid Ligand Framework of Copper(II) with Stable Intrinsic Blue-Light Luminescence in Aqueous Solution

**DOI:** 10.3390/nano11092281

**Published:** 2021-09-02

**Authors:** Suwitra Charoensuk, Jing Tan, Mohini Sain, Hathaikarn Manuspiya

**Affiliations:** 1 The Petroleum and Petrochemical College, Chulalongkorn University, Bangkok 10330, Thailand; suwitra.c@gmail.com; 2 Center for Biocomposites and Biomaterials Processing, Department of Mechanical and Industrial Engineering, University of Toronto, Toronto, ON M5S 3B3, Canada; jingbuct.tan@utoronto.ca; 3 Center of Excellence in Petrochemical and Materials Technology, Bangkok 10330, Thailand

**Keywords:** copper coordination complexes, 3D network complexes, crystal structure, electronic structure, luminescence

## Abstract

Single-crystal solid–liquid dual-phase hybrid organic–inorganic ligand frameworks with reversible sensing response facilitated by external stimuli have received significant attention in recent years. This report presents a significant leap in designing electronic structures that display reversible dual-phase photoluminescence properties from single-crystal hybrid ligand frameworks. Three-dimensional Cu(C_3_N_2_H_4_)_4_Cl_2_ complex frameworks were formed through the intermolecular hydrogen bonding and π⋯π stacking supramolecular interactions. The absorption band peaks at 627 nm were assigned to d–d transition showing 10Dq = 15,949 cm^−1^ and crystal field stabilization energy (CFSE) = 0.6 × 10Dq = 114.4 kJmol^−1^, while the ligand-to-metal charge transfer (LMCT) of complexes was displayed at 292 nm. The intense luminescence band results from LMCT present at 397 nm. Considering its structure, air stability, framework forming and stable luminescence in aqueous solution, the Cu(C_3_N_2_H_4_)_4_Cl_2_ complex shows potential for luminescence Cu-based sensors using emission intensity to detect heavy metal ion species.

## 1. Introduction

Over the past decade, the study of copper(II) coordination complexes has been rapidly developing due to their structural variety and wide range of potential applications such as catalysis [1], gas absorption [2], photocatalysis [3], electrocatalytic [4], magnetic [5], perovskite solar cell [6], luminescence [7], etc. The copper(II) ion, paramagnetic d^9^ configuration, enables the generated strong metal–ligand (M–L) bonding through hard–soft acid–base (HSAB) principles [8]. Many copper(II) coordination complex geometry with different coordination numbers have been reported, such as distorted tetrahedral or square planar [9], tetragonal bipyramidal [10], square pyramidal [11] and distorted octahedral [12] according to a typical Jahn–Teller effect [13]. The Jahn–Teller effect strongly results in the electron distribution that affects the arrangement of atoms in crystal structure. Thus, Jahn–Teller distortions prompt formation of copper(II) coordination complexes leading to the different M–L bond geometries mentioned above [14].

Among those of N-donor or base ligands, the N-heterocyclic imidazole (Im) coordination ligands (M–L) have gained considerable attention as the formation of three-dimensional network complex structures through intermolecular hydrogen bonds and π⋯π supramolecular interactions have various applications. The imidazole ligand is generally considered to be a moderate s-donor and weak π-acceptor, as well as strong ligands for copper(II). In the case of tetragonal geometry, imidazole coordinates to copper(II) coupling Cu(C_3_N_2_H_4_)_4_Cl_2_ similar to pyridine with the occupying coplanar position corresponding to metal ion, while the metal–halide (M–X) bond locates on the axial axis of the coordination complex. Chlorotetrakis(imidazole) copper(II) chloride, Cu(C_3_N_2_H_4_)_4_Cl_2_, was synthesized by Li and co-workers [15], using CuCl_2_.2H_2_O and imidazole in methanol, and the obtained crystals can be described as having a monoclinic crystal structure and space group P2_1_/n with unit cell parameters, namely a = 8.8662(3), b = 13.3199(4), c = 13.3190(4) Å and β = 90.0420(10)°, different than the report from Jian et al. [16]. Jian and co-workers reported that the coordination complexes belong to the monoclinic space group P2_1_/c and show different unit cell parameters: a = 13.909(3), b = 8.8933(18), c = 15.086(7) Å and β = 118.32(2)°. The most common way to understand the electronic structure of the copper coordination network is UV–Vis spectra analysis corresponding to the molecular orbital energy diagram. The spectra bands can determine the d–d transition of the metal center atom and charge transfer (CT) in metal and ligand bonding.

This research embarks on the study of the electronic structure of the copper(II) coordination complex and new findings on its luminescent properties for electrocatalytic process with in situ sensing along with other types of sensing in smart materials, such as actuators. Furthermore, we have reported the luminescence of Cu(C_3_N_2_H_4_)_4_Cl_2_ complexes characterized by fluorescent spectroscopy. The results contribute that the orbitals from ligand charge transfer play a crucial role in the origin of luminescence at 397 nm emission wavelength observed by excitation wavelength equal to 330 nm.

## 2. Materials and Methods

### 2.1. Materials

Copper (II) chloride (CuCl_2_.2H_2_O, 99.9%, Aldrich, Saint Louis, MO, USA), imidazole (C_3_N_2_H_4_, 99.5%, Aldrich, Saint Louis, MO, USA), absolute methanol (CH_3_OH, 99%) and formic acid (HCOOH, 99%) were commercially available and used as purchased, without further purification.

### 2.2. Synthetic Procedure of Copper Coordinate Complexes

The synthetic route used here is an adaptation of the previously reported method for the preparation of the (C_3_N_2_H_5_)[Mn(HCOO_3_)] compounds [17] in order to accomplish similar perovskite structure by replacing Mn^2+^ to Cu^2+^. In a typical experiment, 5 mL of methanol solution of imidazole and formic acid were placed at the bottom of vial glass. Upon the HCOOH-C_3_N_2_H_4_ solution, 2 mL of methanol was carefully added, followed by carefully layering 8 mL of CuCl_2_.2H_2_O solution by using mol ratio 3:1:3 of C_3_N_2_H_4_:CuCl_2_.2H_2_O:HCOOH. The vial was sealed and kept undisturbed at 50 °C. The blue crystal appeared after several days. They were collected, washed with ethanol and dried at room temperature. All are air stable. The unexpected reaction was assembled. The obtained copper coordination complexes had not formed structure with formate like (C_3_N_2_H_5_)[Mn(HCOO)_3_] as mentioned above but bonded to imidazole and chloride instead shown as Cu(C_3_N_2_H_4_)_4_Cl_2_ chemical formula. For the (C_3_N_2_H_5_)[Mn(HCOO)_3_] structure, Mn^2+^ as hard metal and therefore form stronger complexes with oxygen donors than N-pyridine, according to the hard–soft acid–base (HSAB) principle. The formate ligands also act as bridging Mn^2+^ and form ABO_3_ perovskite framework and imidazolium ion located in cavities by NH⋯O hydrogen bond interaction. On the other hand, the N-donor of imidazole is the borderline base and prefers to form stronger complexes with borderline acid Cu^2+^, leading to obtain Cu(C_3_N_2_H_4_)_4_Cl_2_ coordination complexes.

### 2.3. Characterizations

#### 2.3.1. Single-Crystal X-ray Crystallography (SC-XRD)

The single-crystals were analyzed with Mo Kα (λ = 0.71073 Å) on a ‘Bruker APEX-II CCD’ diffractometer. The crystals were kept at 273K during data collection. The chemical structure was determined by using the Olex2 program where charge flipping was used, as well as XH refinement packaging, using CGLS minimization.

#### 2.3.2. X-ray Diffraction (XRD)

XRD pattern were collected by using a Rigaku model Smart Lab 4800 diffractometer with a Cu Kα radiation wavelength (λ = 1.54 Å), generated at a voltage of 40 kV and a filament emission of 30 mA. Sample powder was scanned from the 2θ = 10 to 40° range at a scan speed of 2°/min and a scan step of 0.02° at room temperature.

#### 2.3.3. X-ray Photoelectron Spectroscopy

The binding energy of complexes were measured by XPS (Kratos Axis Ultra DLD) with a monochromatic Al Kα as an X-ray source (anode HT = 15 kV) and energy dispersive X-ray analysis (EDX, JSM-7610 F, JEOL, Akishima, Tokyo, JAPAN). The depth of penetration of XPS is 10 nm. Data evaluation was performed by using CasaXPS.

#### 2.3.4. Element Analysis

CHN analysis was performed to confirm the elemental composition: C, H and N by TruSpec Micro CHNS modal. For Cu and Cl composition, the crystal was detected by Electron Probe-Micro analyzer at Earth Science Centre, University of Toronto. Cu(C_3_N_2_H_4_)_4_Cl_2_ was found to be 35.14% C, 3.94% H, 27.32% N, 15.49% Cu and 18.10% Cl, similar to the expected 35.43% C, 3.97% H, 27.55% N, 15.62% Cu and 17.43% Cl.

#### 2.3.5. Fourier Transform Infrared Spectroscopy (FTIR)

Cu(C_3_N_2_H_4_)_4_Cl_2_ coordination crystals were examined by using an ATR Nicolet iD7 FTIR spectrometer. All spectra were scanned between 4000 and 400 cm^−1^, with 128 convolutions at a resolution of 4 cm^−1^.

#### 2.3.6. Thermal Analysis

Thermogravimetric analyses were performed by using a TGA Q500 (TA Instruments, Newcastle, DE, USA). The procedures were conducted under nitrogen with flow rate 60 mL/min, following. Crystal samples of 10–15 mg was placed on a platinum pan, where a temperature range of 25–800 °C was reached, with a heating ramp of 5 °C/min. Samples were studied for mass loss curves.

#### 2.3.7. UV–Vis and Fluorescent Spectrophotometer

The UV–Vis spectra were carried in PerkinElmer, Lambda 35 UV–Vis spectrophotometer in the wavelength window of 200–800 nm. The Cu(C_3_N_2_H_4_)_4_Cl_2_ crystals were dissolved in deionized water and sonicated for 15 min before measuring. Photoluminescence (PL) was measured by using the fluorescent spectrometer (Fluorolog-3, Horiba, Edison, NJ, USA) of solution.

## 3. Results and Discussion

### 3.1. Molecular Structure and Crystallography Data of [Cu(II)(C_3_N_2_H_4_)_4_Cl]Cl, or [Cu(II)Im_4_Cl]Cl, Coordination Framework

Hybrid organic–inorganic ligand frameworks of [Cu(II)(C_3_N_2_H_4_)_4_Cl]Cl were synthesized and analyzed by element analysis and single-crystal and powder X-ray diffractions. The results of the chemical reaction showed that imidazole was protonated then formed square pyramidal C_4v_ of [CuIm_4_Cl]^+^ coordination complexes similar to pyridine ligands [18]. According to our results, crystallography data of [Cu(II)(C_3_N_2_H_4_)_4_Cl]Cl shows that complexes crystallize in space group P2_1_/n monoclinic system with similar unit cell parameters to those reported by Li et al. [15] and Otieno et al. [19]. The unit cell parameters of the obtained crystals were a = 8.8707(4), b = 13.3224(8), c = 13.925(6) Å, α, γ = 90 and β = 90.094(2). Table 1 exhibits all the crystallography data of [Cu(II)(C_3_N_2_H_4_)_4_Cl]Cl, a hybrid organic–inorganic ligand complex which crystallizes in space group P2_1_/n monoclinic system similar to those in previous works. SC-XRD revealed that the blue plate crystals, [Cu(II)(C_3_N_2_H_4_)_4_Cl]Cl, exhibit a square pyramidal coordination of Cu(II) as metal center atom. Four equatorial N atoms of imidazole ligands were linked by copper(II) to form a base of a square pyramid, and copper(II) also bonded one of the chlorine atoms perpendicular to the plane located at the apex of the pyramid.

**Figure 1:**
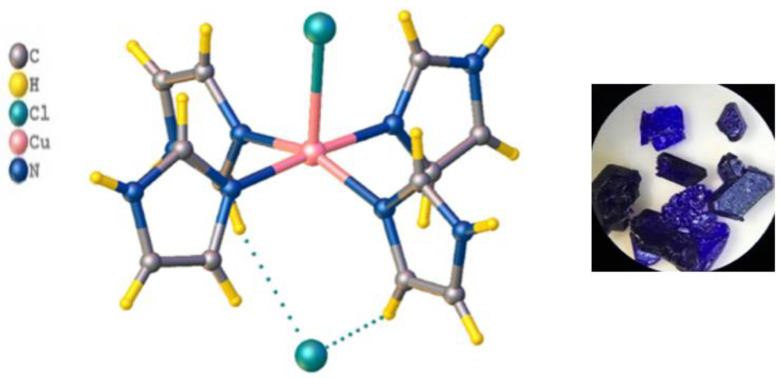
Coordination environment about the Cu(II) metal center of square pyramidal [Cu(II)(C_3_N_2_H_4_)_4_Cl]Cl coordination complex showing hydrogen bonding as dashed line and its optical image.

The square pyramid weakly bound axial chlorides as counterions through hydrogen intermolecular interactions [15]. There are two weak hydrogen bonds, C1-H1⋯Cl(2) and C9-H12⋯Cl(2), which link the discrete [Cu(II)(C_3_N_2_H_4_)_4_Cl]^+^ cation and Cl^-^ counter anion in an asymmetrical unit as presented in the crystal structure illustrated in Figure 1.

**Figure 2:**
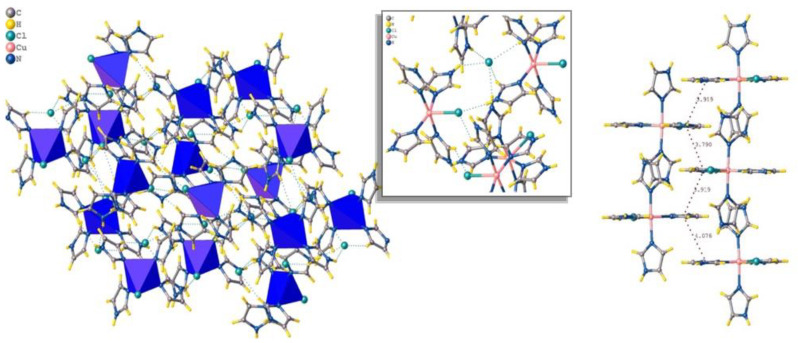
A continuous 3D of copper hybrid organic–inorganic ligand frameworks showing the intermolecular interactions of hydrogen bonding and π⋯π stacking.

The intermolecular hydrogen bonds consequently resulted in a connected three-dimensional network with N-H⋯Cl and C-H⋯Cl interactions (Figure 2). Another significant 3D network, forming the π⋯π supramolecular interactions between neighboring imidazole ligands, plays a role in the packing system, as shown in Figure 2. The π⋯π interactions were reported by measuring the distance between one centroid and the next centroid in an imidazole ring (Ct⋯Ct). All geometry parameters of intermolecular interactions including angles and distances are displayed in Supplementary Materials Table S1.

**Figure 3:**
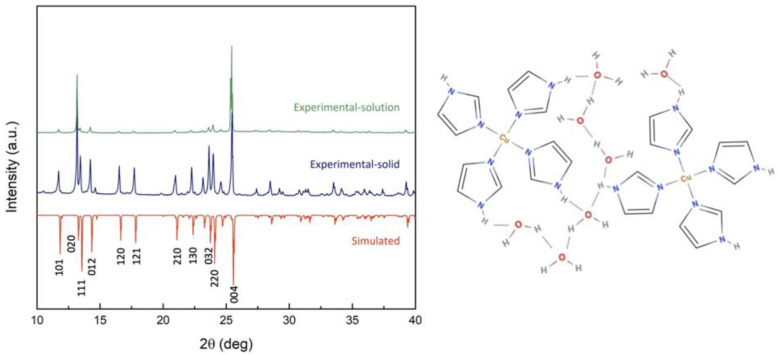
Simulated and experimental XRD patterns of copper(II) coordination complexes (**left**) and hydrogen bond interaction between coordination complexes and water molecules (**right**).

Furthermore, the crystal structure of the [Cu(II)(C_3_N_2_H_4_)_4_Cl]Cl complexes were reversed their crystal structure as can be seen from powder X-ray diffraction results. The simulated XRD patterns of coordination complexes can be obtained by using CIF file data from SC-XRD analysis and then simulating via Vista software. Both experimental and simulated XRD patterns show similar characteristic diffraction peaks as shown in Figure 3. Thus, the single crystalline phase of synthesized coordination complexes is confirmed.

The XRD patterns of aqueous solution of [Cu(C_3_N_2_H_4_)_4_Cl]Cl coordination complexes were shown in same pattern compared with the solid pattern in Figure 3. It reveals that the [Cu(C_3_N_2_H_4_)_4_Cl]Cl complexes can be stable in aqueous surrounding due to intermolecular hydrogen bond between water molecules and HN-imidazole rings, as shown in Figure 3.

### 3.2. Electronic Structure of [Cu(II)(C_3_N_2_H_4_)_4_Cl]Cl Coordination Complexes

**Scheme 1:**
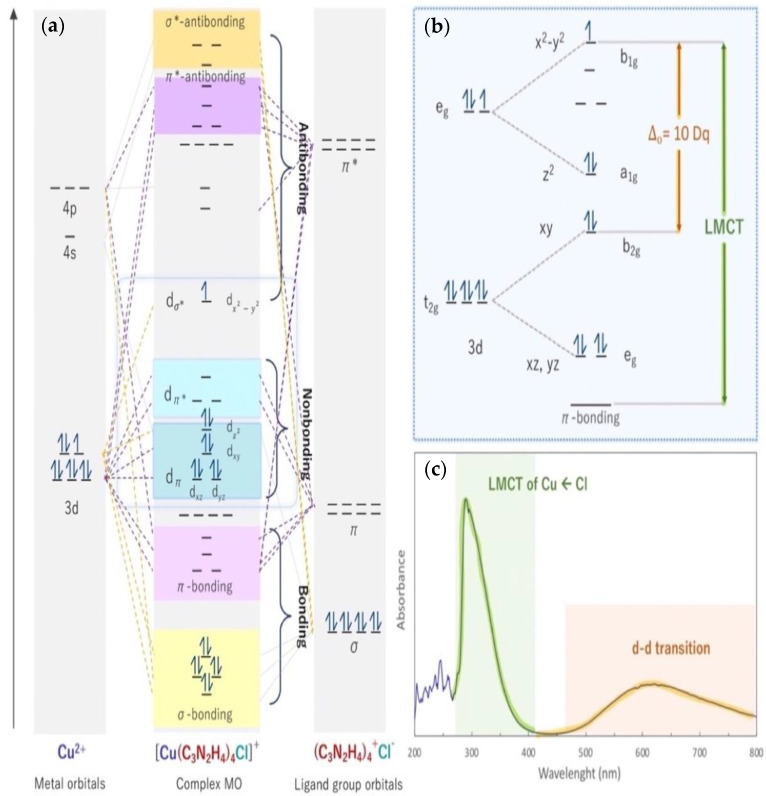
The molecular orbital diagram showing ground state and excited state (*) (**a**), the Cu^2+^ electronic configuration 3d^9^ (t_2g_^6^ e_g_^3^) (**b**) and UV−Vis spectra (**c**) of copper hybrid organic−inorganic ligand frameworks.

The transition metal of Cu^2+^ (paramagnetic d^9^ configuration) and the imidazole ligand could potentially form strong metal–ligand (M–L) bonding through HSAB principles [8]. The N-donor of imidazole was the borderline base and therefore form stronger complexes with borderline acid Cu^2+^. The square pyramidal copper coordination networks consisting of five-coordinate ligands in this work are not Jahn–Teller active, due to an undegenerated occupied d-orbital in their geometries [20]. The ligand field depiction (Scheme 1) implies similar dσ- and σ-levels of copper metal and imidazole ligand, respectively, which can generate Cu-L coordinate bonding as σ-bonds. The single occupancy of the dx^2^-y^2^ (Cu^2+^) orbital coupling to the lone pair electron on the nitrogen of the imidazole labeled as σ-donor and the paired electrons of the antibonding of dz^2^ (Cu^2+^) result in increased antibonding electron density along the axial axis of Cu-Cl. It is noted that the apical elongation length of Cu-Cl does not conform to the Jahn–Teller effect.

Besides σ-bonding, π-bonding is another important interaction in coordination networks. Furthermore, the couplings of d(xy) (Cu^2+^) orbitals with ring π-orbitals and nitrogen π -donors are shown in Scheme 1. Apparently, the Chloride anion (Cl^−^) labeled as the π -donor ligand may donate additional electron to the d-orbital of the π-acceptor (Cu^2+^) generating Cu-Cl coordinate bonding in proper symmetric geometry. The UV–Vis spectroscopy of coordination complex was presented in Scheme 1. The electronic spectra of complexes were obtained from solute sample dissolved with water. The Cu^2+^ electronic configuration 3d^9^ (t_2g_^6^ e_g_^3^), which undergoes the Jahn–Teller effect in an octahedral crystal field, agrees with the distortion octahedral geometry. The blue solution of copper(II) coordination complexes in aqueous solution shows a broad absorption band in the visible range of the spectrum at 627 nm which represents d–d transitions assignable to ^2^E_g_ → ^2^T_2g_ based on a Cu^2+^ central ion (Scheme 1). According to the discrete cation of coordination complexes, [Cu(II)(C_3_N_2_H_4_)_4_Cl]^+^, d–d transition shows 10Dq = 15,949 cm^−1^ and crystal field stabilization energy (CFSE) = 0.6 × 10Dq = 114.4 kJmol^−1^ [21]. Other absorption bands in the UV region are assigned to ligand-to-metal charge transfer (LMCT) from the highest energy ligand-localized molecular orbital to the half-occupied highest energy copper(II) d–d molecular orbital [22]. The absorption bands are expected to undergo electron transition of intra-ligand π → π * and ligand-to-metal charge transfer (LMCT): n_(Imidazole)_ → Cu(II), π_1(Imidazole)_ → Cu(II), π_2(Imidazole)_ → Cu(II) and π_Chloride_ → Cu(II). As seen in Scheme 1, The intense absorption band at 292 nm exhibits LMCT transition of Cl(π) → Cu(dx^2^-y^2^) [23,24] resulting from the presence of a single Cu-Cl coordination bond in aqueous solution.

### 3.3. Valent State and Binding Energy of Copper Coordination Complexes

**Figure 4:**
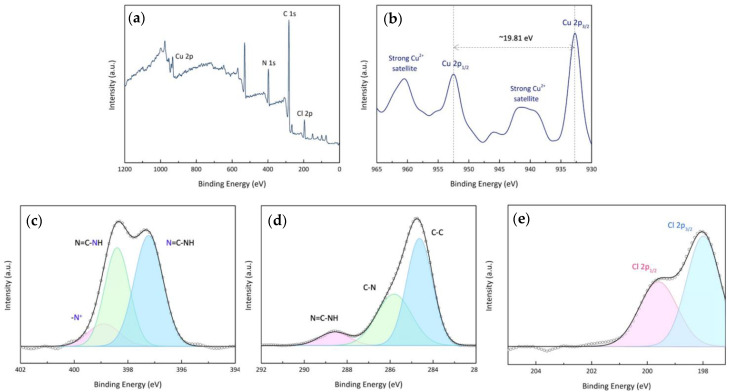
XPS spectra of Cu(C_3_N_2_H_4_)_4_Cl_2_ showing wide spectrum and binding energy states in (**a**) wide scan, (**b**) Cu 2p, (**c**) C1s, (**d**) N1s and (**e**) Cl2p orbitals.

X-ray photoelectron spectroscopy (XPS) of [Cu(II)(C_3_N_2_H_4_)_4_Cl]Cl complex powder was carried out to designate the valence states and support the presence of bonding of copper coordination complexes and [Cu(II)(C_3_N_2_H_4_)_4_Cl]^+^ ions. The wide spectrum shown in Figure 4 indicates all elements in the coordination complexes, i.e., C, N, Cl and Cu. The highest intensity of C 1s shows three peaks due to different carbon species at 284.65, 285.84 and 288.59 eV that correspond to C=C, C-N and C-N bonding, while the N1s peak at 397.22 eV is assigned to C=NH-C which is the pyridine-type (N_py_) in imidazole ring as the position of the basal plane of the square pyramidal. The C-NH-C or the pyrrole-type interaction was observed at the binding energy of 398.42 eV [25,26]. In addition, the presence of peak at 398.91 eV belongs to the positively charged nitrogen (-N^+^), which originates from the pyridine-type nitrogen with the lone pair electrons transfer to Cu center atom. As shown in Figure 4, two strong peaks at 931.9 and 951.8 eV are consistent with the literature data on Cu 2p_3/2_ and Cu 2p_1/2_, respectively [27]. These peaks can be attributed to the Cu^2+^ state in the [Cu(II)(C_3_N_2_H_4_)_4_Cl]^+^ ion. The states are different around 20 eV, which is close to the value reported in the literature [27]. The observed shakeup satellite peaks at 942 and 963 eV are correspond to the paramagnetic chemical state of Cu(II) ion [27,28]. The binding energy of Cl 2p were obtained by deconvolution of line shape. Two Gaussian fitted peaks at 198 and 200 eV can be assigned to Cl 2p_3/2_ and Cl 2p_1/2_, respectively. The almost half-intense Gaussian peak of Cl 2p_1/2_ with respect to Cl 2p_3/2_ also supported the fact that the Cl atoms are present in only one π-bonding of Cu-Cl along the axial axis and another Cl atom act as counter anion to stabilize charge of [Cu(C_3_N_2_H_4_)_4_Cl]^+^ [28].

### 3.4. FTIR, Element Analysis and TGA Thermogram

The existence of imidazole ligands was confirmed by FTIR analysis. The strong bands in the range of 3500 to 2500 cm^−1^ signify the hydrogen bond interactions in the crystals. The N-H⋯Cl intermolecular interaction can be observed at 3289 cm^−1^ corresponding to N-H stretching vibration of the imidazole ring to chloride [29], while the 1492 cm^−1^ band corresponds to the intermolecular hydrogen bond of N-H bending vibration in the solid state. As seen in Figure 3, the C-H stretching vibrations were assigned at 3116 and 2949 cm^−1^. The N-H bending vibrations were assigned to the imidazole ring coupled from C=C (1533 cm^−1^) and C=N (1425 cm^−1^) stretching vibrations resulting in the overtone band at 2846 cm^−1^ as well [30]. The strong band at 1066 cm^−1^ corresponds to C-N stretching vibration. In the copper complexes, C-H (out-of-plane) vibrations generate bands mainly at 862, 794 and 773 cm^−1^, while N-H (out-of-plane) vibrations show at 613 cm^−1^. From FTIR spectrum, Cu-ligands’ vibrations shown at 659 cm^−1^ result from the torsion vibrations of imidazole rings [30].

**Figure 5:**
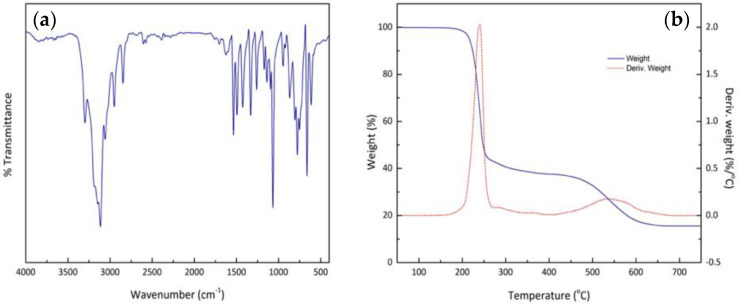
(**a**) FTIR spectra showing functional group characteristic peaks at different wavenumbers, and (**b**) TGA thermogram of Cu(C_3_N_2_H_4_)_4_Cl_2_ coordinate complexes.

Further, the existence of imidazole as ligands in coordination complexes was investigated by thermogravimetric analysis, as shown in Figure 5. The TGA thermogram revealed the decomposition profile with two-step losses. The first weight loss appeared in the range of temperatures from 200 to 250 °C and was caused by a major loss of organic component, namely imidazole (C_3_N_2_H_4_) [31]. At higher temperature, the second-step decomposition is possibly the cause of deformation of inorganic components; copper and chloride undergo 85% weight loss at 650 °C [32]. It is noted that the remaining residues as shown in the tail of weight loss after the second step may correspond to the decomposition products present in the materials [33]. The element analysis was carried out by using the CHN analyzer to obtain the percentage amounts of carbon, nitrogen and hydrogen in coordination complexes.

Then, The EMPA has allowed to determine the copper and chloride composition of coordination complexes. The elemental composition of the coordination complex is given as C, 35.14; H, 3.94; N, 27.32; Cu, 15.49; and Cl, 18.10%.

### 3.5. Fluorescence Spectra

**Figure 6:**
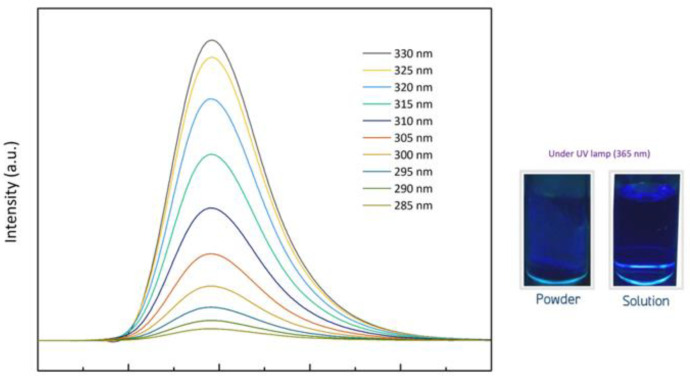
Emission spectra of copper coordinate complexes at each excitation wavelength.

The fluorescence spectra of [Cu(II)(C_3_N_2_H_4_)_4_Cl]Cl coordination complexes were measured by a fluorescence spectrometer at ambient temperature. The synthesized complex was grounded and then treated by deionized water for 30 min in an ultrasonic bath to get the uniform aqueous suspension. The solution of complexes showed high stability in aqueous environment which is confirmed by XRD pattern as describe in Figure 3. Upon excitation wavelength at 330 nm, the complex exhibits the strongest luminescence emission peak at 397 nm and gradually decreases in luminescent intensity when operating lower excitation wavelengths, as shown in Figure 6.

In addition, the observed emission peak is contributed from the π → π * transition of the ligand charge transfer [34]. Considering its structure, air stability, framework forming and stable luminescence in aqueous solution, the Cu(C_3_N_2_H_4_)_4_Cl_2_ complex shows potential for luminescence Cu-based sensors. The emission intensity quenching and enhancing behavior can be further studied for selective solvent or heavy metal ion in aqueous environment and developed to various applications

## 4. Conclusions

In summary, the Cu(II) square pyramidal coordination complex based on imidazole and chloride ligands was synthesized and studied on crystal and electronic structure levels. This copper(II) coordination complex Cu(C_3_N_2_H_4_)_4_Cl_2_ geometry is not subject to the Jahn–Teller active. The intermolecular interaction of hydrogen bonds and π⋯π supramolecular interactions play a significant three-dimensional network-forming of a coordination complex. The absorbance spectra of complexes were obtained from aqueous solution of [Cu(II)(C_3_N_2_H_4_)_4_Cl]Cl coordination complex in the wavelength range of 200–800 nm. The absorption band peaks at 627 nm were assigned to d–d transition, showing 10Dq = 15,949 cm^−1^ and crystal field stabilization energy (CFSE) = 0.6 × 10Dq = 114.4 kJmol^−1^, while the ligand-to-metal charge transfer (LMCT) of complexes displayed at 292 nm. The intense luminescence band results from the ligand-to-metal charge transfer present at 397 nm. The results contribute that the ligand charge transfer not only can reveal the presence Cu-Cl coordination in aqueous solution but also plays a crucial role in the origin of luminescence at 397 nm emission wavelength. According to their structure, air stability, framework forming and stable luminescence in aqueous solution, the Cu(C_3_N_2_H_4_)_4_Cl_2_ complex shows potential for luminescence Cu-based sensors. The emission intensity quenching and enhancing behavior can be further studied for selective solvent or heavy metal ion in aqueous environment. Furthermore, the result described herein will be approached to new insight Cu^2+^-containing hybrid ligand frameworks which are enable for novel multifunctional metal-based system in material sciences.

## Figures and Tables

**Table 1:** Crystallography data for Chloridotetrakis(imidazole)copper(II) chloride, [Cu(II)(C_3_N_2_H_4_)_4_Cl]Cl.

Crystallography Data	
Empirical formula	C_12_H_16_N_8_Cl_2_Cu
Formula weight	406.77
Temperature/K	273.15
Crystal system	monoclinic
Space group	P2_1_/n
a/Å	8.8707(4)
b/Å	13.3224(8)
c/Å	13.9250(6)
α/°	90
β/°	90.094(2)
γ/°	90
Volume/Å^3^	1645.64(14)
Z	4
ρ_calc_g/cm^3^	1.6417
μ/mm^−1^	1.663
F(000)	830.7
Crystal size/mm^3^	0.42 × 0.4 × 0.32
Radiation	Mo Kα (λ = 0.71073)
2Θ range for data collection/°	5.44 to 61.14
Index ranges	−10 ≤ h ≤ 12, −19 ≤ k ≤ 6, −19 ≤ l ≤ 15
Reflections collected	7881
Independent reflections	4439 [R_int_ = 0.0395, R_sigma_ = 0.0566]
Data/restraints/parameters	4439/0/217
Goodness-of-fit on F^2^	1.072
Final R indexes [I ≥ 2σ (I)]	R_1_ = 0.0359, wR_2_ = 0.0868
Final R indexes [all data]	R_1_ = 0.0520, wR_2_ = 0.0959
Largest diff. peak/hole/e Å^−3^	0.58/−0.62

## Data Availability

The data are available upon reasonable request from the corresponding author.

## Supplementary Materials

The following are available online at https://www.mdpi.com/article/10.3390/nano11092281/s1. All crystallography data and CIF file of crystals are shown in the supplementary information. Table S1. Geometry intermolecular interaction for [Cu(II)(C3N2H4)4Cl]Cl coordination complexes. Table S2. Bond Lengths for Chloridotetrakis(imidazole)copper(II) Chloride. Table S3. Fractional Atomic Coordinates (×104) and Equivalent Isotropic Displacement Parameters (Å2 × 103) for Chloridotetrakis(imidazole)copper(II). Ueq is defined as 1/3 of the trace of the orthogonalised UIJ tensor. Table S4. Anisotropic Displacement Parameters (Å2 × 103) for Chloridotetrakis(imidazole) copper(II) Chloride. The Anisotropic displacement factor exponent takes the form: −2π2[h2a*2U11+2hka*b*U12+…]. Table S5. Bond Angles for Chlo-ridotetrakis(imidazole)copper(II) Chloride. Table S6. Hydrogen Atom Coordinates (Å × 104) and Isotropic Displacement Parameters (Å2 × 103) for Chloridotetrakis(imidazole)copper(II) Chloride. Table S7. Presenting all functional groups of Cu(C3N2H4)4Cl2 via different wavenumbers.
