# Supplementary material for: A Single Crystal Hybrid Ligand Framework of Copper(II) with Stable Intrinsic Blue-Light Luminescence in Aqueous Solution
Source: Nanomaterials (Basel). 2021 Sep 2;11(9):2281. doi: 10.3390/nano11092281 (PMC8471168; doi:10.3390/nano11092281)
Supplement: Supplementary file 1 [file nanomaterials-11-02281-s001.zip › nanomaterials-1341889-Supplementary materials.pdf]

nanomaterials

Article

A Single Crystal Hybrid Ligand Framework of Copper(II) with Stable Intrinsic Blue-Light Luminescence in Aqueous Solution

Suwitra Charoensuk [1], Jing Tan [2], Mohini Sain [2,*] and Hathaikarn Manuspiya [1,3,*]

[1] The Petroleum and Petrochemical College, Chulalongkorn University, Bangkok 10330, Thailand; suwitra.c@gmail.com
[2] Center for Biocomposites and Biomaterials Processing, Department of Mechanical and Industrial Engineering, University of Toronto, Toronto, ON M5S 3B3, Canada; jingbuct.tan@utoronto.ca
[3] Center of Excellence in Petrochemical and Materials Technology, Bangkok 10330, Thailand
* Correspondence: m.sain@utoronto.ca (M.S.); hathaikarn.m@chula.ac.th (H.M.)

Citation: Charoensuk, S.; Tan, J.; Sain, M.; Manuspiya, H. A Single Crystal Hybrid Ligand Framework of Copper(II) with Stable Intrinsic Blue-Light Luminescence in Aqueous Solution. *Nanomaterials* **2021**, *11*, 2281. https://doi.org/10.3390/nano11092281

Academic Editors: Sotirios Baskoutas and Marcin Runowski

Received: 30 July 2021
Accepted: 26 August 2021
Published: 2 September 2021

Publisher's Note: MDPI stays neutral with regard to jurisdictional claims in published maps and institutional affiliations.

Copyright: © 2021 by the authors. Licensee MDPI, Basel, Switzerland. This article is an open access article distributed under the terms and conditions of the Creative Commons Attribution (CC BY) license (http://creativecommons.org/licenses/by/4.0/).

The addition of crystallography data was shown in Table below.

Table S1. Geometry intermolecular interaction for [Cu(II)($C_3N_2H_4$)$_4$Cl]Cl coordination complexes.

Hydrogen bonds				
D–H··A	D–H	H··A	D··A	D–H··A
C1–H1··Cl(2)	0.93004(4)	2.785(3)	3.646(3)	154.36(6)
N2–H3··Cl(2)	0.87000(3)	2.530(3)	3.180(3)	133.00(3)
N4–H7··Cl(2)	0.80000(4)	2.460(4)	3.249(4)	169.00(4)
C9–H12··Cl(2)	0.93006(5)	2.768(3)	3.654(3)	159.79(6)
N8–H14··Cl(2)	0.86000(3)	2.401(2)	3.246(2)	167.29(6)
N6–H11··Cl(1)	0.85999(4)	2.439(2)	3.257(2)	159.07(6)
C10–H13··Cl(1)	0.93003(4)	2.781(3)	3.540(3)	139.47(5)
C11–H15··Cl(1)	0.93004(3)	2.859(3)	3.781(3)	171.85(6)
C2–H2··Ct2	0.93004(6)	2.818(3)		
C8–H10··Ct4	0.93000(5)	3.013(3)		

π···π interaction	Ct··Ct Distance
Ct1··Ct3	3.9190
Ct3··Ct3	3.7900
Ct3··Ct1	3.9190
Ct1··Ct1	4.0760

Symmetry codes : H1 = (1/2+x, 1/2-y, +z) ; H2 = (3/2-x, -1/2+y, 3/2-z); H3 = (1+x, +y, +z); H7 = (1/2+x, 1/2-y, 1/2+z); H10 = (2-x, -y, 2-z); H11 = (-1+x, +y, +z); H12 = (1/2+x, 1/2-y, +z); H13 = (1-x, -y, 2-z); H14 = (1/2+x, 1/2-y, -1/2+z); H15 = (-1/2+x, 1/2-y,-1/2+z).

Plane : Ct1, Ct2, Ct3 and Ct4 are the centroids of the imidazole rings N1 C3 N2 C2 C1, N4 C6 N3 C4 C5, C9 N6 C8 C7 N5 and C10 N8 C11 C12 N7, respectively.

Table S2. Bond Lengths for Chloridotetrakis(imidazole)copper(II) Chloride.

Atom	Atom	Length/Å	Atom	Atom	Length/Å
Cu1	Cl1	2.6201(7)	N5	C7	1.366(3)
Cu1	N1	2.016(2)	N5	C9	1.326(3)
Cu1	N3	1.9915(19)	N6	C8	1.358(4)
Cu1	N5	2.009(2)	N6	C9	1.339(3)
Cu1	N7	2.0039(19)	N7	C10	1.319(3)

N1	C1	1.373(3)	N7	C12	1.375(3)
N1	C3	1.312(3)	N8	C10	1.337(3)
N2	C2	1.359(4)	N8	C11	1.356(4)
N2	C3	1.336(3)	C1	C2	1.355(4)
N3	C4	1.381(3)	C4	C5	1.352(4)
N3	C6	1.319(3)	C7	C8	1.354(4)
N4	C5	1.363(4)	C11	C12	1.350(4)
N4	C6	1.343(3)			

Table S3. Fractional Atomic Coordinates (×10[4]) and Equivalent Isotropic Displacement Parameters (Å^2×10^3) for Chloridotetrakis(imidazole)copper(II). U_{eq} is defined as 1/3 of of the trace of the orthogonalised U_{IJ} tensor.

Atom	*x*	*y*	*z*	U(eq)
Cu1	6866.5(3)	2132.6(2)	8774.52(19)	20.81(10)
Cl1	4437.0(7)	1014.3(5)	8790.8(5)	29.97(15)
N1	5856(2)	3487.6(15)	8738.9(14)	22.9(4)
N2	4046(3)	4598.1(18)	8708.2(17)	31.8(5)
N3	6955(2)	2122.1(15)	7345.6(14)	22.5(4)
N4	6679(3)	1743(2)	5826.6(16)	34.0(6)
N5	8572(2)	1139.8(16)	8799.2(14)	23.7(4)
N6	10844(3)	511.4(19)	8741.9(16)	34.9(5)
N7	6956(2)	2209.2(16)	10210.7(14)	24.7(4)
N8	6752(3)	1820(2)	11724.6(15)	34.2(5)
C1	6473(3)	4433(2)	8755(2)	33.1(6)
C2	5356(3)	5127(2)	8736(2)	38.5(7)
C3	4392(3)	3622(2)	8715.6(18)	27.8(5)
C4	7825(3)	2765(2)	6799.8(19)	32.2(6)
C5	7652(4)	2534(2)	5860(2)	35.5(6)
C6	6287(3)	1513(2)	6731.4(17)	27.7(6)
C7	8487(3)	117(2)	8830(2)	33.7(6)
C8	9888(3)	-283(2)	8795(2)	38.8(7)
C9	10028(3)	1357(2)	8750.8(19)	31.1(6)
C10	6408(3)	1552(2)	10825.7(18)	28.8(6)
C11	7540(3)	2692(2)	11695.3(19)	37.9(7)

C12	7670(3)	2927(2)	10755.9(19)	32.9(6)
Cl2	10547.9(7)	4078.0(6)	8749.7(5)	33.25(16)

Table S4. Anisotropic Displacement Parameters (Å2×10[3]) for Chloridotetrakis(imidazole) copper(II) Chloride. The Anisotropic displacement factor exponent takes the form: $-2\pi^2[h^2a^{*2}U_{11}+2hka^*b^*U_{12}+\ldots]$.

Atom	U_{11}	U_{22}	U_{33}	U_{12}	U_{13}	U_{23}
Cu1	24.32(17)	20.86(15)	17.24(15)	4.04(12)	-0.61(12)	0.16(11)
Cl1	26.7(3)	30.2(3)	33.0(3)	-7.1(3)	-0.8(3)	3.0(3)
N1	25.7(11)	20.7(10)	22.3(10)	2.9(8)	2.3(9)	0.8(8)
N2	25.5(12)	30.3(12)	39.6(13)	7.8(10)	0.6(10)	-1.6(10)
N3	25.9(10)	23.2(10)	18.4(9)	2.1(8)	0.5(8)	-0.1(8)
N4	38.5(14)	43.8(15)	19.6(11)	2.5(11)	-3.7(10)	-8.5(10)
N5	24.2(11)	24.0(11)	22.8(10)	3.3(8)	-0.1(9)	-0.4(8)
N6	21.5(11)	44.1(14)	39.0(13)	8.5(10)	2.4(10)	3.4(11)
N7	27.5(11)	26.8(11)	19.9(10)	3.4(9)	-0.7(9)	1.0(8)
N8	31.5(12)	52.1(16)	19.1(10)	1.4(11)	3.0(9)	7.4(10)
C1	27.0(14)	25.2(13)	47.1(17)	-0.9(11)	1.2(13)	2.0(12)
C2	40.3(16)	23.0(13)	52.1(18)	2.4(12)	-3.2(14)	-1.0(13)
C3	24.5(13)	29.8(14)	29.1(13)	-1.0(11)	-0.2(11)	0.4(11)
C4	40.6(16)	27.0(14)	29.1(13)	-3.6(12)	0.7(12)	-0.6(11)
C5	43.7(17)	36.1(15)	26.7(14)	2.7(13)	6.2(13)	3.5(11)
C6	27.2(13)	30.3(14)	25.6(13)	-0.1(11)	-1.5(11)	-5.0(10)
C7	27.7(14)	25.7(13)	47.8(17)	-0.7(11)	0.3(13)	1.0(12)
C8	39.8(16)	27.3(15)	49.4(18)	11.5(13)	-0.1(14)	-0.5(13)
C9	27.6(14)	28.3(14)	37.3(15)	0.2(11)	2.7(12)	2.3(11)
C10	29.1(14)	30.0(14)	27.3(13)	0.1(11)	-1.9(11)	5.4(10)
C11	38.1(16)	54(2)	21.4(13)	-1.5(14)	-2.7(12)	-6.8(12)
C12	36.0(15)	34.0(15)	28.6(13)	-5.9(12)	-2.0(12)	-1.6(11)
Cl2	29.1(3)	43.5(4)	27.2(3)	8.4(3)	-0.1(3)	2.2(3)

Table S5. Bond Angles for Chloridotetrakis(imidazole)copper(II) Chloride.

Atom	Atom	Atom	Angle/°	Atom	Atom	Atom	Angle/°
N1	Cu1	Cl1	98.25(6)	C9	N5	Cu1	126.10(18)
N3	Cu1	Cl1	92.19(6)	C9	N5	C7	105.8(2)
N3	Cu1	N1	89.99(8)	C9	N6	C8	108.5(2)
N5	Cu1	Cl1	104.17(6)	C10	N7	Cu1	126.88(18)
N5	Cu1	N1	157.58(9)	C12	N7	Cu1	127.17(18)
N5	Cu1	N3	88.95(8)	C12	N7	C10	105.9(2)
N7	Cu1	Cl1	92.96(6)	C11	N8	C10	108.5(2)
N7	Cu1	N1	89.76(8)	C2	C1	N1	109.5(2)
N7	Cu1	N3	174.82(8)	C1	C2	N2	105.8(3)
N7	Cu1	N5	89.30(8)	N2	C3	N1	111.2(2)
C1	N1	Cu1	130.07(17)	C5	C4	N3	109.2(2)
C3	N1	Cu1	124.30(18)	C4	C5	N4	106.3(3)
C3	N1	C1	105.6(2)	N4	C6	N3	110.6(2)
C3	N2	C2	107.9(2)	C8	C7	N5	109.9(3)
C4	N3	Cu1	124.63(17)	C7	C8	N6	105.6(2)
C6	N3	Cu1	129.35(18)	N6	C9	N5	110.1(2)
C6	N3	C4	106.0(2)	N8	C10	N7	110.3(2)
C6	N4	C5	108.0(2)	C12	C11	N8	105.8(2)
C7	N5	Cu1	128.01(18)	C11	C12	N7	109.5(3)

Table S6. Hydrogen Atom Coordinates (Å×10⁴) and Isotropic Displacement Parameters (Å²×103) for Chloridotetrakis(imidazole)copper(II) Chloride.

Atom	*x*	*y*	*z*	U(eq)
H3	3140(30)	4850(20)	8670(20)	36(8)
H7	6320(40)	1490(30)	5360(30)	70(13)
H11	11811(3)	477.2(19)	8708.5(16)	41.8(6)
H14	6513(3)	1495(2)	12235.8(15)	41.1(7)
H1	7499(3)	4575(2)	8775(2)	39.7(7)
H2	5463(3)	5821(2)	8741(2)	46.2(8)
H4	3686(3)	3106(2)	8705.5(18)	33.4(7)
H5	8433(3)	3275(2)	7040.3(19)	38.7(7)

H6	8106(4)	2852(2)	5341(2)	42.6(8)
H8	5635(3)	996(2)	6901.3(17)	33.2(7)
H9	7598(3)	-251(2)	8869(2)	40.5(7)
H10	10144(3)	-960(2)	8804(2)	46.6(8)
H12	10425(3)	2002(2)	8726.7(19)	37.3(7)
H13	5861(3)	982(2)	10658.1(18)	34.6(7)
H15	7915(3)	3053(2)	12215.0(19)	45.5(8)
H16	8165(3)	3488(2)	10515.0(19)	39.4(7)

Table S7. Presenting all functional groups of Cu(C3N2H4)4Cl2 via different wavenumbers.

Wavenumber (cm^{-1})	Functional Group
3289	N-H stretching
3116, 2949	C-H stretching
2846	N-H bending
1533	C=C stretching
1425	C=N stretching
1066	C-N stretching
862, 794, 773	C-H (out-of-plane)
613	N-H (out-of-plane)
659	Cu-ligands
